# A prospective phase I dose-escalation trial of stereotactic ablative radiotherapy (SABR) as an alternative to cytoreductive nephrectomy for inoperable patients with metastatic renal cell carcinoma

**DOI:** 10.1186/s13014-018-0992-3

**Published:** 2018-03-20

**Authors:** Rohann J. M. Correa, Belal Ahmad, Andrew Warner, Craig Johnson, Mary J. MacKenzie, Stephen E. Pautler, Glenn S. Bauman, George B. Rodrigues, Alexander V. Louie

**Affiliations:** 1 Department of Radiation Oncology, London Regional Cancer Program, London, Canada; 2 Department of Medical Oncology, London Regional Cancer Program, London, Canada; 3 Division of Urology, Western University, London, Canada; 4 Division of Surgical Oncology, Western University, London, Canada; 5 Department of Epidemiology and Biostatistics, Western University, London, Canada

**Keywords:** Primary tumour, Renal cell, Metastatic, Stereotactic ablative radiotherapy

## Abstract

**Background:**

Cytoreductive nephrectomy is thought to improve survival in metastatic renal cell carcinoma (mRCC). As many patients are ineligible for major surgery, we hypothesized that SABR could be a safe alternative.

**Methods:**

In this dose-escalation trial, inoperable mRCC patients underwent SABR targeting the entire affected kidney. Toxicity (CTCAE v3.0), quality of life (QoL), renal function, and tumour response (RECIST v1.0) were assessed.

**Results:**

Twelve patients of mostly intermediate (67%) or poor (25%) International Metastatic Renal Cell Carcinoma Database Consortium (IMDC) prognostic class, median KPS of 70%, and median tumour size of 8.7 cm (range: 4.8–13.8) were enrolled in successive dose cohorts of 25 (*n* = 3), 30 (*n* = 6), and 35 Gy (n = 3) in 5 fractions. SABR was well tolerated with 3 grade 3 events: fatigue (2) and bone pain (1). QoL decreased for physical well-being (*p* = 0.016), but remained unchanged in other domains. SABR achieved a median tumour size reduction of − 17.3% (range: + 5.3 to − 54.4) at 5.3 months. All patients progressed systemically and median OS was 6.7 months. Crude median follow-up was 5.8 months.

**Conclusions:**

In non-operable mRCC patients, renal-ablative SABR to 35 Gy in 5 fractions yielded acceptable toxicity, renal function preservation, and stable QoL. SABR merits further prospective investigation as an alternative to cytoreductive nephrectomy.

**Trial Registration:**

ClinicalTrials.gov NCT02264548. Registered July 22 2014 – Retrospectively registered: https://clinicaltrials.gov/ct2/show/NCT02264548

**Electronic supplementary material:**

The online version of this article (10.1186/s13014-018-0992-3) contains supplementary material, which is available to authorized users.

## Background

Metastatic renal cell carcinoma (mRCC) represents one of the few clinical scenarios in which randomized evidence supports aggressive primary tumour control via cytoreductive nephrectomy (CN), as this is associated with improved overall survival when followed by interferon-based systemic therapy [1, 2]. Likewise, non-randomized evidence in the era of tyrosine kinase inhibitors (TKI) also supports a survival benefit of CN in select mRCC patients [3, 4] although recent evidence supports superior overall survival with up-front TKI and deferred CN vs. immediate CN [5]. However, rates of CN have declined in the TKI era [6, 7]; moreover, poor-PS and poor-risk patients do not benefit from CN [4] as a result of greater post-surgical morbidity [8].

Stereotactic ablative radiotherapy (SABR) is emerging as an effective ablative modality for renal tumours. Although RCC is considered resistant to standard fractionation radiotherapy [9], hypofractionated radiotherapy yields histologic tumour ablation [10, 11], and clinically, SABR is highly effective in treating RCC oligometastases and can postpone or obviate the need for systemic therapy in some instances [12, 13]. Likewise, SABR is also an emerging ablative modality for primary RCC tumours, achieving high rates of local control with minimal toxicity [14, 15]. In a recent multi-institutional pooled analysis conducted by the International Radiosurgery Oncology Consortium for Kidney (IROCK), 223 patients were treated with SABR targeting RCC primary tumours, yielding a local control rate of 97.8% at 2 years with CTCAE ≥ Gr. 3 toxicity rate of 1.3% [16]. While alternative ablative therapies such as cryotherapy or radiofrequency ablation can target primary tumours, these are limited by size (< 4 cm) and location (peripheral) [17, 18].

We hypothesized that SABR may represent an alternative ablative modality for those patients who may otherwise have benefited from cytoreductive nephrectomy, but are medically inoperable or have unresectable primary tumours. Moreover, given recent evidence that SABR may stimulate anti-tumour immunity to increase sensitivity to contemporary immunotherapy [19–21], there is strong rationale to establish the safety of this technique, permitting its combination with immune checkpoint inhibitors to potentially enhance their effectiveness.

We previously published our retrospective experience [22] wherein SABR safely and tolerably treated unconventionally large renal tumours (median 9.5 cm, above the concensus 5–8 cm upper limit of size [23]). Here we report the results of a prospective trial of 5-fraction SABR targeting the tumour-bearing kidney in mRCC. The primary endpoint was safety/toxicity and secondary endpoints included renal function, patient-reported quality of life, tumour response, and overall survival.

## Methods

### Patient Eligibility

In this ethics board-approved protocol (Western University REB# 15680), eligible patients were ≥ 18 years old, were diagnosed with stage IV, biopsy-confirmed RCC, and were deemed medically inoperable or harbored an unresectable primary tumour. Operability was assessed by a urologic oncologist (all patients were reviewed in a multi-disciplinary clinic including a surgeon, radiation oncologist, and medical oncologist). Patients could be enrolled regardless of systemic therapy status and could receive SABR before or after. Enrolled patients underwent detailed pre-screening including baseline performance status and QoL assessment, bloodwork, renal perfusion scan, and CT imaging. Those with bilateral renal involvement, poor baseline renal function (creatinine clearance < 50 mL/min or < 40% flow to the contralateral kidney on renal perfusion scan), non-RCC histology, or life expectancy < 8 weeks were excluded. Patients receiving systemic therapy were included if treatment was discontinued at least 2 weeks prior to SABR. Detailed inclusion and exclusion criteria are provided on ClinicalTrials.gov (NCT02264548).

### Dose Escalation Protocol

**Fig. 1:**
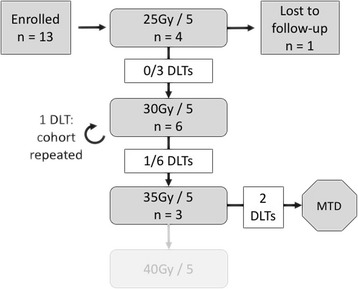
Study Design. DLT – dose limiting toxicity; MTD – maximum tolerated dose

The primary endpoint of this trial was safety/toxicity, evaluated via the dose escalation cohort schema depicted in Fig. 1. Each dose cohort was monitored during treatment and in follow-up for dose limiting toxicities (DLTs), defined as any CTCAE v 3.0 grade 3, 4, or 5 toxicity event that was definitely/probably/possibly related to treatment. Recruitment of at least 3 patients to each dose level (25, 30, 35, and 40 Gy in 5 daily fractions) was planned. Advancement to the subsequent dose level was permitted in the absence of a DLT up to 4 weeks post-SABR. If a DLT event occurred in a cohort, that dose level was repeated with 3 additional patients. If no further DLTs occurred in the repeat cohort, recruitment to the next dose level was permitted. The maximum tolerated dose (MTD) was reached if a second DLT occurred.

Due to a single DLT that occurred in the 30 Gy cohort, a repeat cohort at that dose level was recruited. MTD for this trial was reached when two DLT events were reported in the 35 Gy cohort. Thus, no patients were recruited to the 40 Gy cohort.

### Treatment Technique

All enrolled patients were immobilized in a supine position using a vacuum body mould as per institutional standards. Motion control was achieved using four-dimensional CT simulation wherein images were obtained at all phases of the respiratory cycle to generate a composite integrated target volume. OARs were contoured (without expansion for planning risk volume (PRV)) and dose limited to these structures based on established constraints [23, 24]. The clinical target volume (CTV) included the primary tumour and ipsilateral kidney to recapitulate a cytoreductive nephrectomy. The initial planning target volume (PTV-I) was defined as the CTV with a 5 mm expansion in all three dimensions, whereas the final PTV (PTV-F) was a compromised PTV generated by subtracting any small bowel OAR volume from the PTV-I (Additional file 1: Figure S1). Dose was prescribed to the PTV-F.

### Follow-up and Evaluation of Clinical Outcomes

Patients were monitored for adverse events on each treatment day, then at 4, 8, and 12 weeks, and subsequently at 6-month intervals thereafter. Follow-up visits included clinical assessment of toxicity using the Common Terminology Criteria for Adverse Events version 3.0 (CTCAE v4.0), QoL (FACT-G & FACT-KSI) and general symptom assessment (ESAS) using standard questionnaires, bloodwork (CBC, electrolytes, urea, creatinine, calcium, albumin, liver enzymes, bilirubin, alkaline phosphatase) and urinalysis. Pre- and post-SABR creatinine clearance (CrCl) was calculated using the Cockroft-Gault equation and glomerular filtration rate (GFR) measured using nuclear medicine renal perfusion scans. Post-SABR CT scans were performed at 4, 8, and 12 weeks and then at 6 month intervals post-SABR to assess tumour response.

### Statistical Analysis

Descriptive statistics were generated for baseline patient and disease characteristics. The change in creatinine CrCl, GFR, or QoL scores pre- and post-SABR were evaluated using the Wilcoxon signed rank test. A Kaplan-Meier estimate of overall survival including 95% confidence bands was generated for all patients. All statistical analysis was performed using SAS version 9.4 software (SAS institute, Cary, NC), using 2-sided statistical testing at the 0.05 significance level.

## Results

### Patient and Disease Characteristics

From July 2009 to June 2016, 13 patients were enrolled in the trial beginning at the 25 Gy dose level and proceeding as depicted in Fig. 1. One patient from the 25 Gy cohort was lost to follow-up prior to the first post-treatment visit and thus was excluded. No acute toxicities were recorded for this patient while on treatment. As summarized in Table 1, the remaining 12 patients were of median age 66.8 years (range: 55–85), median KPS of 70% (40–90%), and were mostly of ‘Intermediate’ (66.7%) or ‘Poor’ (25%) International Metastatic Renal Cell Carcinoma Database Consortium (IMDC) prognostic class [25]. The majority of tumours were T2-T4 (91.7%), biopsy-proven RCC (clear cell histology in 75%), and 8.7 cm median size (4.8–13.8). One patient’s tumour histology was “poorly differentiated carcinoma” but was deemed eligible given the clinical context. Median CTV and PTV-F were 535.0 cm^3^ (288.2–883.5) and 763.1 cm^3^ (264.9–1234.4), respectively. Distant metastases occurred most commonly in lung, bone, and brain. Six of the 12 patients received systemic therapy pre-SABR (1), post-SABR (4), or both (1). Systemic therapy was initiated any time after SABR (given a minimum 2 week wash-out period) as indicated for progression of metastatic disease. Additional file 2: Figure S2 depicts the timing of systemic therapy, per patient, relative to completion of SABR. Six patients did not receive systemic therapy.

**Table 1:** Patient Characteristics (*N* = 12)

Characteristic	Value
Age at diagnosis – median (range)	66.8 (55.0–85.1)
Male – n (%)	7 (58.3)
Laterality – n (%)	
Left Kidney	4 (33.3)
Right Kidney	8 (66.7)
Histology – n (%)	
Clear Cell	9 (75)
Papillary	2 (16.7)
Other^a^	1 (8.3)
Location of Metastases^b^	
Lung	10 (83.3)
Bone	6 (50)
Lymph nodes	5 (41.7)
Brain	3 (25)
Other^c^	4 (33.3)
Karnofsky Performance Status – median (range)	70 (40–90)
Karnofsky Performance Status – n (%)	
≥ 80%	5 (41.7)
< 80%	7 (58.3)
IMDC Prognostic Group – n (%)	
Favorable	1 (8.3)
Intermediate	8 (66.7)
Poor	3 (25)
Systemic Therapy Timing^b^ – n (%)	
None	6 (50)
Pre-SABR	1 (8.3)
Post-SABR	4 (33.3)
Both	1 (8.3)
Systemic Therapy Type – n (%)	
None	6 (50)
Pazopanib or Sunitinib	5 (41.7)
Temsirolimus or Everolimus	2 (16.7)
SABR Treatment Technique – n (%)	
VMAT	7 (58.3)
TOMO	5 (41.7)
Dose Cohort (Gy) – n (%)	
25/5	3 (25)
30/5	6 (50)
35/5	3 (25)
Primary tumor size^d^ (cm) – median (range)	8.7 (4.8–13.8)
Actuarial median follow-up (months)^e^ - median (95% CI)	22 (4.63, N/A)

*IMDC*: International Metastatic Renal Cell Carcinoma Database Consortium, *SABR*: stereotactic ablative radiotherapy, *CI*: confidence interval, *TOMO*: helical tomotherapy, *IMRT*: intensity modulated radiotherap. ^a^Poorly-differentiated carcinoma (n = 1); ^b^Categories not mutually-exclusive; ^c^Kidney (n = 1), liver (*n* = 2), left adrenal (n = 1); ^d^Longest tumor dimension on CT scan; ^e^Reverse Kaplan-Meier Method

### Treatment Parameters

Doses to treatment volumes and OAR are listed by individual patient in Additional file 3: Table S1. Favorable coverage of treatment volumes was achieved, with ≥95% of the CTV and PTV-F receiving 95% of the prescribed dose in all trial patients. The PTV-I (median 798.9 cm^3^, 315.1–1265.5) was compromised by a median (range) of 4.0% (0.02–17.9) to exclude small bowel. The median V95 for the PTV-I was 97.5% (80.1–99.9). Plans were optimized such that OAR doses were limited to commonly-accepted constraints for 5-fraction abdominal SABR [23, 24] as listed in Additional file 3: Table S2.

### Toxicity

Patients were recruited to dose cohorts according to an *a priori* dose-escalation scheme (Fig. 1). Demographic information, disease characteristics, and treatment details are listed by patient in Table 2. Overall, treatment was safe and well-tolerated. Table 3 lists all reported toxicity events that were possibly, probably, or definitely treatment-related. Three grade 3 events (possibly, probably, or definitely treatment-related) were reported: 2 fatigue (probably and definitely related) and 1 bone pain (possibly related). A large proportion of grade 1 & 2 toxicities were gastrointestinal, including altered taste, nausea, and vomiting (reported in 75% of patients). Fatigue ≥ grade 1 was reported in 50% of patients. All toxicities were effectively managed with supportive measures. There were no grade 4 or 5 treatment-related events. Patient 9 suffered an unrelated grade 4 pneumonia and patient 6 succumbed to unrelated grade 5 dyspnea secondary to pulmonary embolus.

**Table 2:** Summary of patient and disease characteristics by individual patient (N = 12)

Characteristic	Median^d^	Patient											
		1	2	3	4	5	6	7	8	9	10	11	12
Age	66.8	69	59	85	54	57	61	81	70	76	82	61	64
T Stage	–	T3c	T2a	T3b	T2b	T3c	T2b	T2a	T4	T2a	T2a	T3a	T1b
N Stage	–	N0	NX	N0	NX	N1	NX	N1	N1	N1	N0	N1	N0
Laterality	–	L	R	R	R	R	R	R	R	R	L	L	L
Tumor Size (cm)^a^	8.7	9.3	7	8.9	11.9	11.2	13.8	7.3	10	7.9	7.5	9.8	4.8
Radiation Dose (Gy) / Fractions	–	25/5	25/5	25/5	30/5	30/5	30/5	30/5	30/5	35/5	30/5	35/5	35/5
∆ CrCl (mL/min.)	−13.5	−13.5	35.9	−12.1	−4.6	NR	−48.2	NR	NR	−36.6	−24.7	−54	17.5
Local Response (%)^b^	−17.3	−11.8	−17.1	−30.3	−25.2	−25.9	−5.8	NR	NR	−54.4^c^	+ 5.3	−17.5	−14.6
Time to Systemic Progression (months)	3.9^d^	4.34	0.7	10.9	20.5	0.6	0.9	0.8	0.5	12.2	15.0	5.2	3.9
Systemic Therapy	–	P	P	–	P,E	–	T	–	–	–	–	S	S
Survival (months)	6.7^d^	4.9	10.2	16.4	43.6	1.5	3.2	1.3	2.4	13.6	22^e^	6.7	4.6

*L*: left kidney, *R*: right kidney, *HT* – helical tomotherapy, *IMRT*: intensity modulated radiotherapy, *VMAT*: volumetric arc therapy, *NR*: not reported, *CrCl*: Creatinine clearance, **∆** - change, *P*: pazopanib, *E*: everolimus, *T*: temsirolimus, *S*: sunitinib. ^a^Longest tumor dimension on CT scan; ^b^Maximal reduction (−) or increase (+) in primary renal tumor size following SABR, expressed as percent of initial size; ^c^Pt. 9 also underwent renal embolization (post-SABR) for refractory hematuria; ^d^Median overall survival and time to systemic progression calculated using Kaplan-Meier method (95% CIs: 1.48–16.43 and 0.7–10.7, respectively); ^e^Alive at last follow-up

**Table 3:** All Possible Treatment-Related Toxicity Events^a^ (N = 12)

Toxicity	Grade 1	Grade 2	Grade 3
Nausea	13	3	
Vomiting	4	1	
Altered Taste	4		
Anorexia		1	
Gas		2	
Diarrhea		2	
Constipation	1		
Dermatitis	2	2	
Fatigue	2	10	2
Pain - Abdominal	5		
Pain - Bone	1		1
Weakness	2	1	
Lower Limb Edema	4		
Urinary^b^	2		
Insomnia	2		
Neuropathy	1		
Hot flashes	3		
Dizziness		1	
Headache	3		

^a^Expressed as no. of events using CTCAE v3.0 and including both acute (on treatment) and late (reported in follow-up ≥4 weeks post-SABR) ^b^Frequency & urgency

### Renal Function

**Fig. 2:**
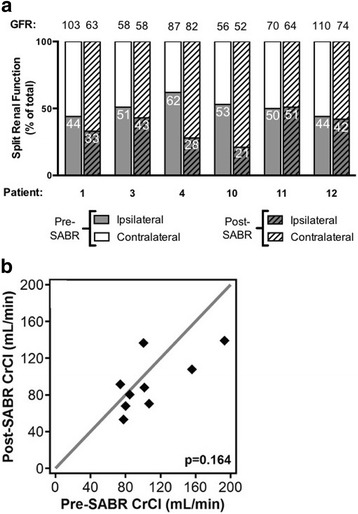
Renal Function. **a** Functional contribution of both kidneys pre- and 12 weeks post-SABR with corresponding measured GFR values listed above each bar. Numbered bars indicate % contribution from ipsilateral kidney. **b** Comparison of creatinine clearance (CrCl) calculated using the Cockroft-Gault equation before and after stereotactic ablative radiotherapy (SABR) for patients with available pre- and post-SABR data (*n* = 9). *P*-value reported from the Wilcoxon signed-rank test

Overall renal function was largely preserved following renal SABR. Nuclear medicine scans revealed reductions in the proportion of function of the ipsilateral kidney as well as in measured GFR in most patients (Fig. 2a). No significant reduction in GFR was observed at 12 weeks post-SABR [median 72.7 (range: 56.0–109.9) vs. 62.8 (52.3–74.04) mL/min/1.73m^2^, *p* = 0.125]. Likewise, pre- and post-SABR (at last follow-up) creatinine clearances were not significantly different [median 98.1 (range: 40.1–193) vs. 88.1 (53.1–139) mL/min, *p* = 0.164] (Fig. 2b). No patients required dialysis.

### Quality of Life (QoL)

Patient-reported quality of life was assessed by Functional Assessment of Cancer Therapy (FACT) questionnaires at baseline (*n* = 12) and at scheduled follow-up visits (*n* = 10). QoL declined with respect to physical well-being (*p* = 0.016), but remained unchanged in the functional, social and emotional domains as well as overall QoL (*p* > 0.05). Likewise, QoL scores for a kidney-specific index (FACT-KSI) were similar pre- and post-SABR (*p* > 0.05). Results are summarized in Additional file 3: Table S3.

### Tumour Response and Overall Survival

**Fig. 3:**
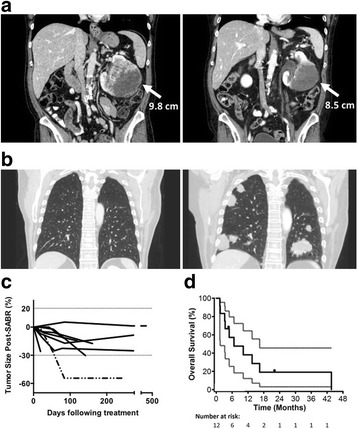
Treatment Response and Survival. **a** Abdominal and **b** thoracic coronal CT slices from patient 11 pre-SABR (left) and 7 months post-SABR (right). **c** Tumour size expressed as percent change in longest tumour dimension. Horizontal dotted lines mark a 20% increase and 30% decrease in size. Patient 9 (dotted/dashed line) also underwent renal embolization for refractory hematuria on day 27 post-SABR. **d** Kaplan-Meier plot of overall survival with 95% confidence bands (*n* = 12)

Despite local disease stability with a median tumour size reduction of − 17.3% (range: + 5.3 to − 54.4) at a median follow-up time of 5.3 months (95% CI: 1.1–9.4), all patients progressed systemically (Fig. 3c). Overall survival (95% CI) at 6, 12, and 24 months was 57.1% (25.4–79.6), 38.1% (12.1–64.4), and 19.1% (3.0–45.6), respectively (Fig. 3d). Median (95% CI) overall survival was 6.7 months (1.5–16.4). Crude median follow-up (range) was 5.8 months (1.4–43.6).

## Discussion

Here we report on the first prospective trial of renal-ablative SABR as an alternative to cytoreductive nephrectomy in non-operable patients with mRCC. Building on our institutional experience of this technique in a case-series of unconventionally large renal tumours [22], this phase I trial evaluated cytoreductive SABR targeting the whole ipsilateral kidney, in essence to recapitulate the effects of debulking nephrectomy.

Our key findings include the safe delivery of 35 Gy in 5 fractions with minimal toxicity, favorable renal outcomes, preserved quality of life, and local disease stability in all cases where longitudinal imaging was available. Our trial design deliberately employed a conservative fractionation schedule so as to limit dose to organs at risk and thus minimize toxicity in this metastatic patient population with poor PS. A five-fraction regimen is in keeping with multi-fraction SABR protocols which often use 3–5 fractions [16, 23]. With respect to renal function, while no statistically significant decline was observed post-SABR, decreases in mean CrCl and GFR were noted (− 10 mL/min and − 9.9 mL/min/1.73m^2^, respectively). These differences are relatively mild in the context of estimated decline in eGFR following radical nephrectomy: − 22.4 mL/min/1.73m^2^ based on a recent systematic review and meta-analysis (27 articles; 3719 patients) [26].

We hypothesized that primary renal SABR as an alternative to cytoreductive nephrectomy holds promise for several reasons. First, as tyrosine-kinase inhibitors (TKIs) are considered the standard of care in mRCC, the initiation of these agents would be delayed following a nephrectomy owing to surgical healing. In contrast, SABR is a convenient non-invasive outpatient procedure that, in other disease sites, requires only a short delay (i.e. 2 weeks) prior to the initiation of a pharmacologic agent. In fact, one prospective study suggests that SABR to an extra-renal target with concomitant pazopanib is safe [27]. Second, many primary targets in mRCC are technically inoperable due to tumour size or proximity to the collecting vessels and ureter. SABR can be targeted precisely to access any location within the affected kidney and dose can be sculpted within organ-at-risk tolerance. Third, SABR is inherently tissue-sparing, whereas a nephrectomy may result in decline of pre-existing renal dysfunction or even *de novo* chronic kidney disease.

Finally, SABR holds promise for mRCC particularly in the era of contemporary immunotherapy. Despite the excitement surrounding checkpoint inhibition, response rates appear to reach a ceiling of 20–40% [28]; for nivolumab, which has been approved for second-line treatment of mRCC, the response rate is 25% [29]. Thus, it is essential to explore strategies to extend the benefits of these agents to additional patients. SABR has emerged as one such strategy since its mechanism of action partly involves inciting anti-tumour immune activation [30, 31] as well as abscopal (out-of-field) effects in mRCC and other malignancies [32, 33] - a phenomenon also thought to involve systemic anti-tumour immunity [19–21]. Interestingly, PD-1 inhibition can increase the frequency of radiotherapy-induced abscopal effects in a xenograft mouse model of RCC [20]. Clinically, several case reports have emerged demonstrating improved systemic responses when radiotherapy and checkpoint inhibition are combined [34–37]. Singh *et al.* recently demonstrated local anti-tumour immune activation in renal tumours following single-fraction SABR [38]. Our demonstration of the safety and tolerability of SABR in mRCC therefore justifies the combination SABR with immune checkpoint inhibition as a rational therapeutic strategy worthy of further investigation.

The limitations of this study include its small sample size at a single institution. Also, given the short median OS and consequently short follow-up in this trial (crude median follow-up: 5.8 months), the full range and severity of late toxicities may be under-appreciated. Another limitation is the heterogeneity in systemic treatment among the patients on this trial: specifically, the agents used and their timing was variable. Moreover, 50% of patients were not fit enough to receive systemic therapy. The OS of 6.7 months were relatively short and probably reflect, in part, the heterogeneity in patient selection and treatment; more careful patient selection and uniform treatment would likely be associated with better survival outcomes. Finally, it is unclear what the optimal local response assessment tool is in this setting, as distinguishing response from post-treatment effect remains challenging. The strengths of this study are its prospective design with *a priori* dose-escalation scheme and its novelty; to our knowledge, no other institution has evaluated post-SABR outcomes in the mRCC setting as we have done here.

## Conclusion

We observed low toxicity with SABR to a dose of 35Gy in 5 fractions targeted to the primary tumour and ipsilateral kidney. Exploration of this regimen as an alternative to CN in larger prospective trials with up-front cytoreductive SABR followed by systemic therapy appears feasible based on our results. Additionally, tumour-targeted SABR could allow further dose-escalation while sparing normal renal parenchyma. Given the hypothesized immune activation induced by SABR, combining renal SABR with immune checkpoint inhibition in mRCC could be a particularly interesting approach and a multi-institution phase II trial is planned.

## Additional files

Additional file 1: **Figure S1.** Example of treatment planning volumes. To facilitate treatment of large PTVs, the initial PTV (teal outline) was trimmed in areas of overlap (green colorwash) with organs at risk - particularly small bowel (purple colorwash) – trimmed to generate a final PTV (red colorwash). (JPEG 127 kb)

Additional file 2: **Figure S2.** Clinical Course by Individual Patient. Time from initial radiologic diagnosis to death or last follow-up for each patient (*y*-axis intersect: SABR completion date). Duration and type of systemic therapy, additional local therapies, and date of systemic progression are indicated. ^1^Palliative embolization of bulky pelvic metastasis followed by hip replacement surgery; ^2^Palliative radiotherapy (20 Gy/5) following hip replacement; ^3^Renal artery embolization for refractory hematuria; ^4^Palliative radiotherapy to painful bony metastasis (shoulder). (JPEG 111 kb)

Additional file 3: **Table S1.** Target Dose and Volume Data by Individual Patient. CTV- clinical target volume; PTV-I – initial planning target volume; PTF-F – final planning target volume; NR – not reported. **Table S2.** Organ at Risk (OAR) Constraints and Doses (cGy). D1–99% of the contoured volume receives this dose or less; D33–67% of the contoured volume receives this dose or less; *Small bowel dose constraints were exceeded in Patients 2 & 6. Patient 2 reported no toxicity whatsoever and Patient 6 reported only grade 1 emesis. **Table S3.** Summary of Quality of Life (QoL) Data at Baseline and Last Available Follow-Up (*N* = 12*). (DOCX 28 kb)

## Abbreviations

CN: Cytoreductive nephrectomy
CrCl: Creatinine clearance
CTCAE: Common Terminology Criteria for Adverse Events
eGFR: Estimated glomerular filtration rate
GFR: Glomerular filtration rate
Gy: Gray
IMDC: International Metastatic Renal Cell Carcinoma Database Consortium
IROCK: International Radiosurgery Oncology Consortium for Kidney
PRV: Planning risk volume
PS: Performance status
RCC: Renal cell carcinoma
SABR: Stereotactic ablative radiotherapy
