# Supplementary figures and images for: A prospective phase I dose-escalation trial of stereotactic ablative radiotherapy (SABR) as an alternative to cytoreductive nephrectomy for inoperable patients with metastatic renal cell carcinoma
Source: Radiat Oncol. 2018 Mar 20;13:47. doi: 10.1186/s13014-018-0992-3 (PMC5859400; doi:10.1186/s13014-018-0992-3)

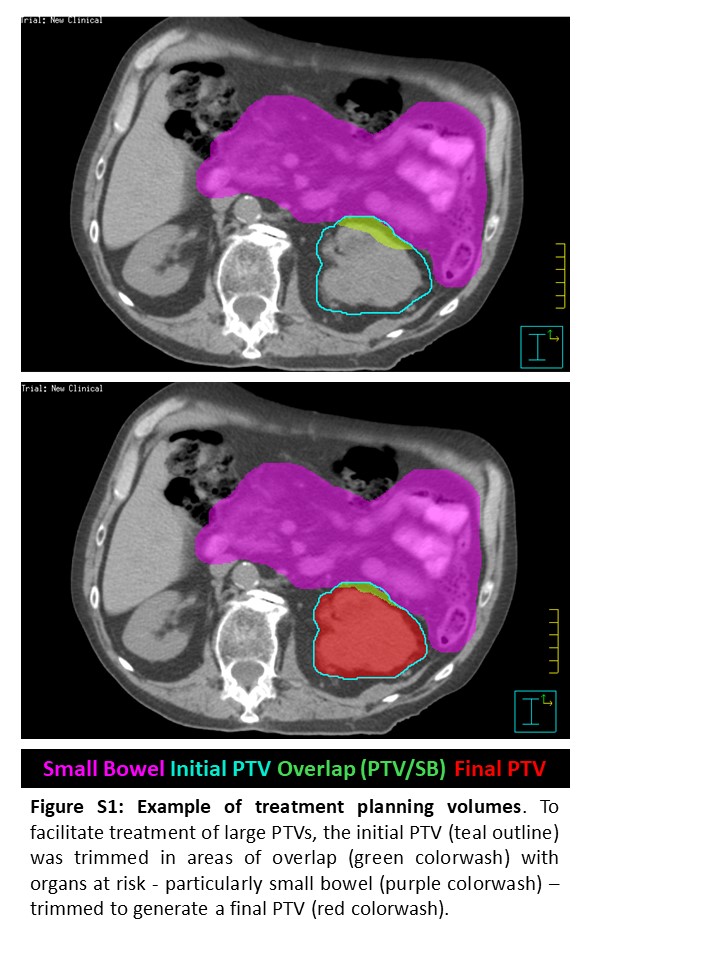

Supplement: Supplementary file 1 — Figure S1. Example of treatment planning volumes. To facilitate treatment of large PTVs, the initial PTV (teal outline) was trimmed in areas of overlap (green colorwash) with organs at risk - particularly small bowel (purple colorwash) – trimmed to generate a final PTV (red colorwash). (JPEG 127 kb) [file 13014_2018_992_MOESM1_ESM.jpg]

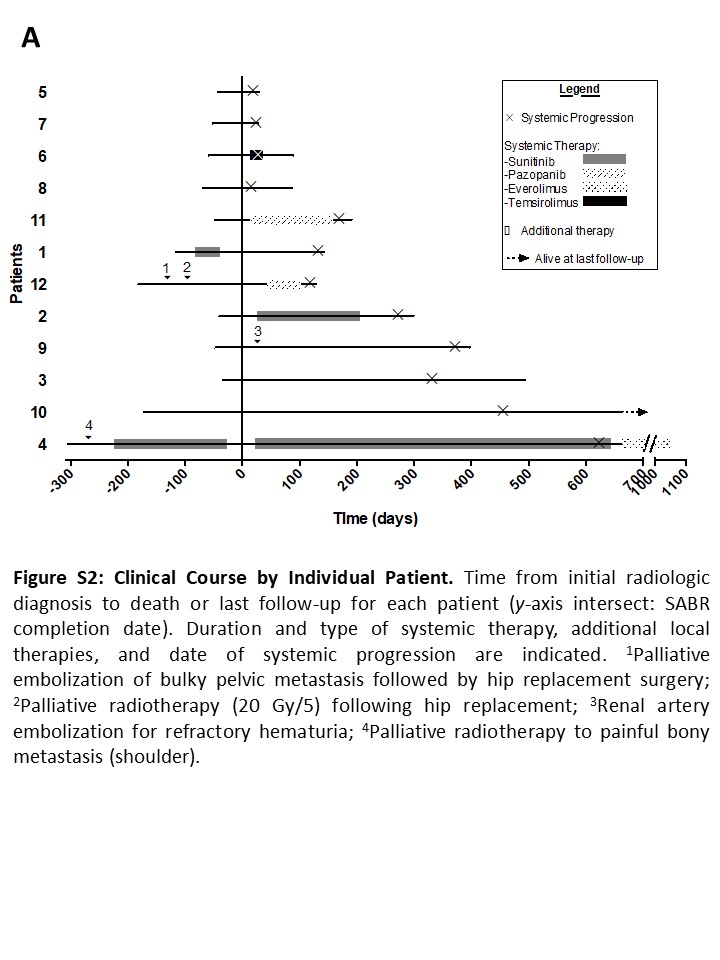

Supplement: Supplementary file 2 — Figure S2. Clinical Course by Individual Patient. Time from initial radiologic diagnosis to death or last follow-up for each patient (y-axis intersect: SABR completion date). Duration and type of systemic therapy, additional local therapies, and date of systemic progression are indicated. 1Palliative embolization of bulky pelvic metastasis followed by hip replacement surgery; 2Palliative radiotherapy (20 Gy/5) following hip replacement; 3Renal artery embolization for refractory hematuria; 4Palliative radiotherapy to painful bony metastasis (shoulder). (JPEG 111 kb) [file 13014_2018_992_MOESM2_ESM.jpg]
