# Supplementary material for: A prospective phase I dose-escalation trial of stereotactic ablative radiotherapy (SABR) as an alternative to cytoreductive nephrectomy for inoperable patients with metastatic renal cell carcinoma
Source: Radiat Oncol. 2018 Mar 20;13:47. doi: 10.1186/s13014-018-0992-3 (PMC5859400; doi:10.1186/s13014-018-0992-3)
Supplement: Supplementary file 3 — Table S1. Target Dose and Volume Data by Individual Patient. CTV- clinical target volume; PTV-I – initial planning target volume; PTF-F – final planning target volume; NR – not reported. Table S2. Organ at Risk (OAR) Constraints and Doses (cGy). D1–99% of the contoured volume receives this dose or less; D33–67% of the contoured volume receives this dose or less; *Small bowel dose constraints were exceeded in Patients 2 & 6. Patient 2 reported no toxicity whatsoever and Patient 6 reported only grade 1 emesis. Table S3. Summary of Quality of Life (QoL) Data at Baseline and Last Available Follow-Up (N = 12*). (DOCX 28 kb) [file 13014_2018_992_MOESM3_ESM.docx]

**Table S1: Target Dose and Volume Data by Individual Patient**

| **Parameter** | **Patient Number** | | | | | | | | | | | | **Median** |  |
| --- | --- | --- | --- | --- | --- | --- | --- | --- | --- | --- | --- | --- | --- | --- |
|  | **1** | **2** | **3** | **4** | **5** | **6** | **7** | **8** | **9** | **10** | **11** | **12** |  |  |
| **Treatment technique** | IMRT | IMRT | TOMO | TOMO | TOMO | TOMO | IMRT | VMAT | VMAT | VMAT | TOMO | TOMO | - | |
| **Radiation dose (cGy) / Fractions** | 2500/5 | 2500/5 | 2500/5 | 3000/5 | 3000/5 | 3000/5 | 3000/5 | 3000/5 | 3500/5 | 3000/5 | 3500/5 | 3500/5 | - | |
| **Tumor size (cm)** | 9.3 | 7 | 8.9 | 11.9 | 11.2 | 13.8 | 7.3 | 12.5 | 7.9 | 7.5 | 9.8 | 4.8 | 8.7 | |
| **CTV**  **Volume (cm^3^)**  **V95 (%)** | 543.4  100.0 | 288.2  100.0 | 310.9  99.8 | 692.4  99.6 | 846.3  99.1 | 883.5  98 | 369.8  99.9 | 535.04  96.0 | 357.5  100.0 | 329.2  99.8 | 721.9  99.3 | NR  NR | 535.0  99.8 | |
| **PTV-I**  **Volume (cm^3^)**  **V95 (%)** | 812  99.7 | 549.2  91.4 | 507.9  98.6 | 1265.5  97.5 | 1154.5  97.7 | NR  NR | 577.6  99.1 | 798.9  88.5 | 817.3  99.9 | 528.1  96.1 | 1025.5  94.1 | 315.1  80.1 | 798.9  97.5 | |
| **PTV-I compromised for PTF-F (%)** | 1.4 | 17.9 | 5.5 | 2.61 | 2.4 | NR | 0.6 | 9.2 | 0.02 | 4.1 | 4.0 | 15.9 | 4.0 |  |
| **PTV-F**  **Volume (cm^3^)**  **V95 (%)** | 800.5  95.8 | 450.9  99.7 | 480.2  100.0 | 1232.4  99.5 | 1126.9  99.7 | 1234.4  98.5 | 574.1  99.6 | 725.7  96.9 | 817.1  99.9 | 506.6  99.5 | 984.7  98.8 | 264.9  95.1 | 763.1  99.6 | |

CTV- clinical target volume; PTV-I – initial planning target volume; PTF-F – final planning target volume; NR – not reported.

**Table S2: Organ at Risk (OAR) Constraints and Doses (cGy)**

| **OAR** | **Dose Limit (cGy)** | **Patient Number** | | | | | | | | | | | | **Maximum** |
| --- | --- | --- | --- | --- | --- | --- | --- | --- | --- | --- | --- | --- | --- | --- |
|  |  | **1** | **2*** | **3** | **4** | **5** | **6*** | **7** | **8** | **9** | **10** | **11** | **12** |  |
| **Contralateral Kidney**  **Max. point dose**  **D1** | < 800  < 700 | 423  396 | 346  321 | 526  490 | 646  542 | 722  453 | 683  425 | 548  507 | 153  165 | 657  620 | 645  616 | 739  669 | 600  530 | 739  669 |
| **Small Bowel**  **Max. point dose**  **D1** | < 3000  < 2400 | 2503  2398 | 2506  **2433** | 2504  2385 | 2808  2380 | 2897  2313 | **3034**  **2529** | 2315  2120 | 2479  2318 | 2365  1811 | 2335  1973 | 2655  1881 | 2364  2142 | 3034  2529 |
| **Spinal Cord**  **Max. point dose**  **D1** | < 2500  < 2400 | 982  898 | 1007  989 | 1227  1194 | 2001  1966 | 1909  1820 | 1753  1668 | 1711  1597 | 1065  992 | 1020  887 | 1560  1465 | 1712  1625 | 2014  1885 | 2014  1966 |
| **Liver**  **Max. point dose**  **D33** | < 4500  < 2000 | 549  261 | 2622  29 | 2588  1080 | 3138  1795 | 3116  1613 | 3095  1682 | 3115  116 | 3087  124 | 3681  915 | 695  392 | 1644  375 | 1678  545 | 3681  1795 |
| **Pancreas**  **Max. point dose**  **D33** | < 4500  < 2500 | 2591  1479 | 55  25 | 1517  1000 | 2526  1466 | 2439  1448 | 2810  1544 | 141.2  44.9 | 1823  671 | 2319  1547 | 1946  975 | 3619  2285 | 3305  1661 | 3619  2285 |

D1 – 99% of the contoured volume receives this dose or less; D33 – 67% of the contoured volume receives this dose or less;

*Small bowel dose constraints were exceeded in Patients 2 & 6. Patient 2 reported no toxicity whatsoever and Patient 6 reported only grade 1 emesis.

**Table S3: Summary of Quality of Life (QoL) Data at Baseline and Last Available Follow-Up (N = 12*)**

| **QoL Assessment Tool** | **Scores** – median (range) | | | **p-value** |
| --- | --- | --- | --- | --- |
|  | **Baseline (N = 12)** | **Follow-up (N = 10)** | **Change (N = 10)** |  |
| FACT-G Physical | 15.50 (8.00, 26.00) | 14.00 (6.00, 26.00) | -3.00 (-6.00, 1.00) | **0.016** |
| FACT-G Social | 23.50 (16.33, 28.00) | 22.17 (18.67, 28.00) | -0.08 (-7.00, 7.67) | 0.563 |
| FACT-G Emotional | 14.00 (8.00, 22.00) | 17.00 (7.00, 24.00) | 2.00 (-4.00, 9.40) | 0.080 |
| FACT-G Functional | 11.50 (3.00, 26.00) | 10.50 (6.00, 23.00) | -2.08 (-7.00, 13.20) | 0.418 |
| FACT-G Overall | 61.17 (48.00, 94.60) | 68.08 (40.83, 92.83) | -3.83 (-12.60, 19.43) | 0.432 |
| FACT Kidney Symptom Index | 33.50 (17.00, 56.00) | 34.00 (16.00, 49.00) | -0.50 (-12.00, 5.08) | 0.438 |
| FACT Kidney Symptom Index – Disease Related Symptom | 23.50 (15.00, 34.00) | 20.50 (12.00, 33.00) | -0.50 (-8.00, 2.00) | 0.227 |
